# Design and Evaluation of Ethylene Glycol Monopiperate as a Novel Antihyperlipidemic Prodrug: Synthesis, Antihyperlipidemic Activity, and Pharmacokinetics

**DOI:** 10.1155/jamc/3258325

**Published:** 2026-10-27

**Authors:** Guirong Borijihan, Mimi Liu, Mingming Bao, Zhengkun Sun, Meng He, Youxin Li

**Affiliations:** ^1^ Department of Chemistry and Environment Hohhot Minzu College Hohhot China; ^2^ School of Chemistry sand Chemical Engineering Yan’an University Yan’an China yau.edu.cn; ^3^ School of Chemistry and Chemical Engineering Institute of Mongolian Medicine Chemistry Inner Mongolia University Hohhot China imu.edu.cn; ^4^ Tianjin Key Laboratory for Modern Drug Delivery and High-Efficiency Faculty of Medicine School of Pharmaceutical Science and Technology Collaborative Innovation Center of Chemical Science and Engineering Tianjin University Tianjin China tju.edu.cn

**Keywords:** antihyperlipidemic, ethylene glycol monopiperat epiperine, pharmacokinetics, UPLC

## Abstract

Piperine, a major active component isolated from the traditional Mongolian medicine *Piper longum L.*, exhibits antihyperlipidemic activity but also demonstrates inherent toxicity. In this study, ethylene glycol monopiperate (EGM), a piperine derivative, was synthesized via concentrated H_2_SO_4_‐catalyzed esterification. Its structure was systematically characterized by FTIR, MS, ^13^C NMR, and ^1^H NMR, with a yield of 60% and a purity of 99.2%. Subsequently, the in vitro cytotoxicity of EGM on HepG2 cells was evaluated, and the results indicated that over 90% cell viability was observed at concentrations up to 150 μg/mL. It was also investigated in male Wistar rats, and a sensitive, rapid, and accurate solid‐phase extraction (SPE)–UPLC method was developed and validated for the quantification of EGM in rat plasma. The novel method showed good linearity over the concentration range of 1–16 µg/mL, a limit of detection (LOD) of 1 μg/mL, and high accuracy and precision (relative error [RE%] < 15%, relative standard deviation [RSD%] < 10%). In terms of antihyperlipidemic efficacy, EGM demonstrated excellent performance in reducing total cholesterol (TC), glucose (GLU), and low‐density lipoprotein cholesterol (LDL‐C), as well as elevating high‐density lipoprotein cholesterol (HDL‐C); these effects were notably better than the conventional control drug simvastatin. Pharmacokinetic analysis revealed that the plasma concentration‐time profile of EGM followed a first‐order open one‐compartment model. Calculations using WINNONLIN software (ver. 5.2) showed a half‐life (*t*_1/2_) of 1.46 ± 0.67 h and an area under the curve (AUC_0_–∞) of 7,15,530 ± 522.4 ng h/mL. EGM was stable in plasma over 24 h at room temperature and one month at −20°C, after three freeze–thaw cycles. These results verified that EGM may serve as a promising candidate for an antihyperlipidemic prodrug.

## 1. Introduction

Hyperlipidemia, also known as hyperlipoproteinemia, is a metabolic disorder characterized by abnormal lipid metabolism, resulting in elevated plasma levels of one or more lipids (such as low‐density lipoprotein cholesterol [LDL‐C], total cholesterol [TC], and triglycerides [TG]) [1, 2]. It is a growing public health concern and a significant risk factor for many diseases such as atherosclerosis, diabetes, myocardial infarction, ischemic heart disease, and cerebrovascular diseases [3–5]. With the growing prevalence of hyperlipidemia and its related diseases, there is an increased interest in developing novel therapeutic agents that can regulate TC, TG, LDL‐C, high‐density lipoprotein cholesterol (HDL‐C), and glucose (GLU) levels. In 1992, Wu En et al. demonstrated that an extract from the fruit of *Piper longum L.* exhibited significant antihyperlipidemic effects [6]. Recent studies have further indicated that piperic acid derivatives possess substantial therapeutic potential for hyperlipidemia and could serve as long‐term, nontoxic health remedies for the elderly [7, 8]. Notably, several derivatives isolated from the fruit of *Piper longum L.*, including piperlonguminine, piperine, piperic acid polysaccharide, piperic acid butanediol monoester, and pipernonaline, have exhibited antihyperlipidemic properties [8, 9].

Piperine (1‐piperoylpiperidine) is a natural alkaloid found in species of the genus *Piper genus* such as *Piper nigrum* (black and white pepper) and, in particular, *Piper longum L.* (long pepper), plants that hold a significant place in traditional Mongolian medicine [8, 10]. As an adjuvant in various formulations, piperine shows a broad spectrum of pharmacological and biological activities [10–12]. Recent studies have highlighted piperine’s diverse biological effects, including antipyretic, anticancer, antioxidant, anticonvulsant, antidepressant, antithyroid, antiapoptotic, antimetastatic, anti‐inflammatory, and antihyperlipidemic properties [13–16]. The biological activities of piperine are closely related to its chemical structure, which includes key functional groups such as 1, 2‐methylenedioxyphenyl, a conjugated double‐bond side chain, and a piperidine moiety attached via a carbonyl amide, as shown in the Graphic Abstract. Further exploration of related chemical structures is critical for discovering novel antihyperlipidemic compounds [17–19]. To further clarify which functional groups are critical for antihyperlipidemic effects, structural analysis of its derivatives has been conducted. By analyzing a series of derivatives such as piperlonguminine, piperine, piperic acid polysaccharide, and piperic acid butanediol monoester, it was found that the 1, 2‐methylenedioxybenzene group and conjugated double‐bond side chain play a particularly vital role in mediating the antihyperlipidemic effects of piperic acid analogs. Additionally, previous studies on antihyperlipidemic analogs and in vivo assays indicated that a longer side chain on an amide or ester moiety cannot be tolerated, whereas the 1, 2‐methylenedioxybenzene group is crucial [7, 20]. Esterification with ethylene glycol is a common prodrug strategy to mask phenolic groups, which can improve aqueous solubility, alter absorption profiles, alleviate direct gastrointestinal irritation caused by the parent compound, and reduce the direct cytotoxicity of the compound.

In this study, ethylene glycol monopiperate (EGM), a piperine derivative, was synthesized via concentrated H_2_SO_4_‐catalyzed esterification, and the purity was up to 99.2%. Its structure was characterized by FTIR, MS, ^13^C NMR, and ^1^H NMR. The antihyperlipidemic activity of EGM was demonstrated in male Wistar rats, showing significant reductions in TC, TG, GLU, and LDL‐C levels, alongside a slight increase in HDL‐C. In comparative assessments, EGM showed efficacy comparable to or greater than Simvastatin across the five monitored lipid parameters. In addition, a sensitive, rapid, and accurate solid‐phase extraction–ultraperformance liquid chromatography (SPE‐UPLC) method was established and validated to quantify EGM in rat plasma. Pharmacokinetic analysis revealed that EGM followed first‐order kinetics, modeled by an open one‐compartment system.

## 2. Experimental Procedure

### 2.1. Chemicals and Reagents

Sodium chloride (99%), anhydrous magnesium sulfate (98%), concentrated sulfuric acid (98%), n‐hexane (98%), ethyl acetate (99%), ethylene glycol (98%), and toluene (99.5%) were bought from Tianjin Beilian Fine Chemicals Development Co. Ltd (Tianjin, China). Piperine (99%) was bought from the National Institute for the Control of Pharmaceutical and Biological Products. HPLC‐grade methanol and acetonitrile were from the Fisher Company (China). TG, TC, LDL‐C, GLU, and HDL‐C Assay Kits were purchased from Biosino Bio‐Technology and Science Incorporation.

Ultrapure water was prepared using Milli‐Q water purification equipment (Millipore Elix Essential 5 UV Water). The FTIR spectrum of EGM was recorded at room temperature (RT) using a KBr disc with a Vertex70 instrument (Bruker, Germany). ^13^C NMR and ^1^H NMR spectra were recorded with a Bruker spectrometer (500 MHz). The thin‐layer chromatography (TLC) plate with compounds was visualized by a ZF‐20D Cabinet‐type UV Analyzer (245 nm and 365 nm, Gongyi Yuhua Instrument Co., Ltd). Wistar rats were supplied by the Xing Long Experimental Animal Farms (Haidian District, Beijing, China). All animal experimental procedures were performed following the 1996 “Guidance for the Care and Use of Laboratory Animals” (Institute of Laboratory Animal Resources on Life Sciences, National Research Council, and National Academy of Sciences). The ethical approval number for research involving animals on the laboratory animal platform is HMC‐mouse‐20250309.

### 2.2. Synthesis and Characterization of EGM

The synthesis of EGM involved two key processes: the synthesis of piperic acid and the preparation of EGM from piperic acid (Figure 1). Accordingly, piperic acid was prepared by alkaline hydrolysis of piperine as described by Han et al. [8, 18]. In a typical experiment, piperine (0.11 mol, 30 g), potassium hydroxide (0.95 mol, 53.1 g), and 234 mL of 95% ethanol were mixed and refluxed for 2 h. The reaction progress was monitored by TLC using a mixture of ethyl acetate: n‐hexane (4:6, v/v) as the developing solvent. The reaction mixture was cooled to room temperature upon completion, and the solution was acidified to pH 5 by adding aqueous HCl (1 mol/L) to precipitate piperic acid. The resulting precipitate was filtered under vacuum, recrystallized using 20 mL of ethanol, and obtained as yellow needle‐like piperic acid crystals (790 mg, 98.8% yield) with a melting point of 206°C–208°C, as shown in Figure S1a.

**FIGURE 1:**
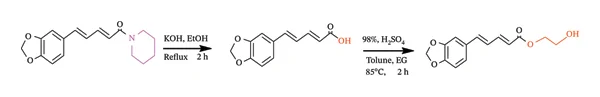
Synthetic process for EGM.

The synthesis of EGM was first reported by Lourenço et al. in 2014, where various synthesis conditions, including catalyst, reaction time, and reaction temperature, were studied to get the optimal yield of EGM. According to this report, the yield could reach 60.18% with p‐toluenesulfonic acid as a catalyst, reflux 5 h at 126°C∼132°C [21]. In this study, we conduct the reaction at 85°C for 2 h with concentrated H_2_SO_4_ as a catalyst. Typically, 20 mL of ethylene glycol and 2 mL of concentrated H_2_SO_4_ (98%) were added to a 100 mL three‐neck round‐bottom flask and stirred at RT for 5–8 min. A 35 mL toluene solution containing 0.08 g/mL of piperic acid was added dropwise, and the mixture was refluxed at 85°C for 2 h. The reaction progress was monitored by TLC under ultraviolet (UV) light at 254 nm and 365 nm, using ethyl acetate: n‐hexane (4:6, v/v) as the developing solvent. After cooling the reaction by holding the flask in ice water, the mixture was extracted twice in a separation funnel with ethyl ether (20 mL, 25 mL) and washed with 10 mL of saturated NaCl solution. The organic layer was dried over anhydrous MgSO_4_ and then concentrated using a rotary evaporator. The crude product (yellow crystals, Figure S1b) was purified by silica gel column chromatography using ethyl acetate: n‐hexane (4:6, v/v) as the mobile phase (MP). Fractions corresponding to the main spot on TLC were collected and concentrated under reduced pressure, then lyophilized to afford the target product as a pale yellow powder, with an isolated yield of 60%. In this esterification reaction, the protonation of the carbonyl group by concentrated H_2_SO_4_ enhanced its electrophilicity, allowing nucleophilic attack by the alcohol group of ethylene glycol. This led to the formation of a tetrahedral intermediate, which then lost a water molecule to form the ester.

### 2.3. UPLC Instrumentation and Operating Conditions

EGM samples were analyzed using a UPLC‐H‐Class system (ACQUITY, Waters, MA, USA) coupled with a quaternary solvent manager, an FTN auto‐sampler, and a photodiode array (PDA) detector. Chromatographic separation was achieved on a UPLC HSS T3 column (ACQUITY, Waters; 2.1 × 100 mm, 1.8 μm) maintained at 35°C. The MP consisted of acidified water and acetonitrile (1:1, v/v), with acidified water prepared by adding 0.1% formic acid to ultrapure water. The flow rate (FR) was set to 0.2 mL/min. A Vanguard precolumn (2.1 × 100 mm) with the same packing material as the analytical column was installed between the analytical column and the injector to intercept the chemical substances and particulates that caused the blockage of the chromatographic column sieve plate and the decrease in column efficiency. Detection was performed at a wavelength of 338 nm. The autosampler temperature was set to 6°C, and an injection volume of 2 μL was used for each sample. Data acquisition and analysis were performed using Empower Software v2.0.

### 2.4. Cell Culture and Cytotoxicity of EGM In Vitro

The in vitro cytotoxicity of EGM on HepG‐2 cells was assessed using the MTT assay. Cells were seeded into 96‐well plates (3K–7K cells/well, 100 μL), followed by incubation at 37°C, 5% CO_2_, and 90% humidity for 24 h, and wells with good growth status, uniform cell distribution, and density were selected for subsequent experiments. Then we replaced the culture medium with EGM sample solutions at various concentrations (30, 50, 100, 150, 200, 250, 300, 350, 400, 450, and 500 μg/mL), and incubated for another 24 h under the same conditions. The growth status of the cells was observed using an inverted microscope, and wells exhibiting good growth, uniform cell distribution, and appropriate density were photographed at 4x/10x magnification for documentation. Subsequently, 10 μL of the MTT solution (5 mg/mL) was added to each well to achieve a final concentration of 0.5 mg/mL, and the plates were incubated for an additional 4 h. Afterward, the EGM‐containing solution was carefully replaced with 100 μL of formazan solubilization solution. The plates were gently shaken at 300 rpm for 10 min to fully dissolve the formazan, and the absorbance of each well was measured (570 nm) in four replicates for each concentration.

### 2.5. In Vivo Evaluation of Antihyperlipidemic Effects

The antihyperlipidemic activity of EGM was evaluated following a previously reported experimental design, as shown in (Figure 2) [8, 10]. Inclusion and exclusion criteria for experimental animals were preset before the initiation of this study. These standards were not modified afterward, and the ARRIVE guidelines were strictly followed throughout the study. Forty male Wistar rats were acclimated to standard laboratory conditions for one week (22 ± 2°C with a 12 h dark‐light cycle). All in vivo experiments were approved by the Animal Care and Use Committee of Hohhot Minzu College (approval No. HMC20250309). Rats weighing 140 ± 10 g with no visible abnormalities following a 7‐day acclimatization period were randomized into four experimental groups (*n* = 10 for each): Control group, HFD, HFD + Simvastatin group, and HFD + EGM group. The control group was fed a standardized chow, while the other groups received a high‐fat diet (HFD) consisting of 45.0% wheat flour, 35.0% corn starch, 7.0% egg yolk, 5.6% wheat bran, 5.0% lard, 1.0% cholesterol, 1.0% NaCl, 0.2% cholic acid, and 0.2% methylthiouracil for 15 days. The rats had free access to water and were fed 20 g of food daily. The dose of EGM was set at 5.6 mg/kg, which was the same as that of piperic acid due to its similar chemical structure [7]. Likewise, the Simvastatin group received the same oral dose (5.6 mg/kg body weight). The control and HFD groups were given an equivalent volume of sodium carboxymethyl cellulose (CMC‐Na, 0.5% in water). All drugs were prepared in a final concentration of 1.4 mg/mL in CMC‐Na (0.5% in water). At the end of the experiment, animals were first deeply anesthetized with an intraperitoneal injection of sodium pentobarbital at a dose of 50 mg/kg body weight. After the loss of the pedal withdrawal reflex and complete unconsciousness, blood was collected via decapitation using a sharp guillotine after 12 h of fasting. The samples were allowed to coagulate and then centrifuged at 1006.2 × g for 15 min at 4°C, and then stored at −20°C until further analysis. Plasma levels of TG, TC, LDL‐C, GLU, and HDL‐C were measured enzymatically using commercially available kits. Throughout the experiment, no animal deaths or invalid serum samples were observed in these four experimental groups. Therefore, the data obtained from all rats were included in the final statistical analysis, with no animals excluded from the dataset.

**FIGURE 2:**
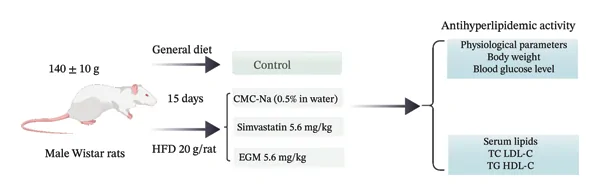
Experimental design for the antihyperlipidemic activity of EGM.

### 2.6. Sample Preparation

#### 2.6.1. Preparation of the Stock Solution (SS), Internal Standard (IS) Solutions, and Working Standards

A 100 μg/mL EGM SS was prepared in acetonitrile. To facilitate the quantitative analysis of EGM in rat plasma, a series of EGM calibrator solutions was prepared by serial dilution of the SS with acetonitrile, resulting in concentrations of 1, 2, 4, 8, 10, 14, and 16 μg/mL. Similarly, three QC samples (limit of quantification [LOQ] at 1, middle QC at 4, and high QC at 14 μg/mL) were prepared. For the IS solution, 100 μL of 1 mg/mL piperine in acetonitrile was prepared. All working solutions were prepared in volumetric flasks and stored at 4°C for future use.

#### 2.6.2. Pretreatment of Plasma Samples

In this study, plasma samples were processed using SPE, employing an Oasis HLB column (1 cc, 30 mg) to purify the plasma samples. The plasma samples spiked with EGM were prepared as follows: 100 μL of EGM standard solutions were placed in centrifuge tubes, and the organic solvent was evaporated using nitrogen gas. Then, 100 μL of plasma and 100 μL of 4% phosphoric acid solution were added to the tubes and vortexed for 1 min. The prepared plasma samples containing spiked EGM were then loaded onto the preactivated SPE column, which had been conditioned with 2 mL of methanol followed by 2 mL of distilled water. The column was washed sequentially with buffer at pH 13.0 and distilled water. Finally, 2 mL of acetonitrile was used to elute EGM under vacuum for 5 min. The eluent was evaporated under nitrogen, reconstituted in 100 μL of acetonitrile, filtered, and 2 μL was injected into the UPLC system for analysis.

To get an optimized treatment strategy, we also performed the liquid–liquid extraction (LLE) method on the pretreatment of the plasma samples. Typically, 100 μL of EGM standard solution was loaded into a 5‐mL centrifuge tube and dried at 45°C under a stream of nitrogen. Afterward, 100 μL blank plasma (thawed at room temperature for 30 min) and 500 μL ethyl acetate were added to the above tube, vortexed for 1 min, and then centrifuged at 10,000 rpm for 15 min. After centrifugation, the supernatant was transferred to a clean vial and dried under nitrogen. The resulting residue was dissolved in 100 μL of acetonitrile and filtered through 0.22 μL syringe filters. Finally, 20 μL of the filtered solution was injected into the UPLC system for further analysis. Each experiment was tested in triplicate. As shown in the Supporting Figure S2, the chromatographic signals obtained using SPE were superior to those from the LLE method, indicating that SPE was an effective approach for the pretreatment of EGM plasma samples.

All experimental procedures were conducted in a dark room to prevent sample isomerization caused by light exposure.

### 2.7. Method Development

For the pharmacokinetic analysis of EGM in rat plasma, UPLC was selected due to its ability to provide precise and reliable results. Different MPs and FRs were tested to optimize the chromatographic conditions and ensure appropriate retention time, sharp peaks, and avoidance of interference from miscellaneous peaks.

### 2.8. Method Validation

The UPLC method was validated for linearity, selectivity, precision, accuracy, recovery, and stability before its application for actual sample analysis. It was worth noting that the stability studies of EGM were performed in rat plasma under a variety of storage and processing conditions. For this validation, 80 μL of piperine IS solution was added to 5‐mL centrifuge tubes along with the EGM standard solution.

To assess selectivity and specificity, blank plasma samples, EGM standards containing the IS, and a real plasma sample taken 15 min after the intragastric administration of EGM were analyzed using UPLC. The linearity was evaluated using seven concentrations of EGM ranging from 1 to 16 g/mL, and the calibration curve was constructed by linear regression with the least‐squares method, applying a weighting factor of 1/*x* ^2^. According to the FDA guidance for bioanalytical method validation, the LOQ was defined as the lowest concentration on the calibration curve that complied with both the linear regression equation and acceptable accuracy/precision requirements. For the developed method, the LOQ was determined to be 1.0 g/mL, where the analyte exhibited a signal‐to‐noise ratio (S/N) of approximately 10, along with an accuracy of 2.6% and precision (expressed as % relative standard deviation, %RSD) of 9.76%—both of which satisfied the predefined acceptance criteria for bioanalytical method validation. To determine the limit of detection (LOD) of EGM, a series of serially diluted biological standard solutions at concentrations of 0.2, 0.4, 0.6, 0.8, and 1.0 μg/mL were prepared following the procedure described in the Sample Preparation section and analyzed by UPLC. The LOD of EGM was determined to be 0.2 μg/mL with an S/N ratio of approximately 3.

Accuracy and precision were determined by calculating the interday and intraday (over 5 days) variation and accuracy for QC samples at 1, 4, and 14 g/mL concentrations, with five replicates for each concentration. The relative recovery and extraction recovery of the bioassay method were also evaluated using QC samples at the same concentrations and replicates. The concentrations of QC samples were calculated from the calibration curve. The accuracy, generally expressed as the relative error (RE%), was calculated by subtracting the nominal concentration from the measured concentration and presenting it as a percentage of the nominal concentration. The precision, described as RSD%, was obtained by dividing the standard deviation of the measured concentration by the mean of the measured concentration, expressed as a percentage.

To evaluate the extraction recovery, QC samples and standard solution at three concentration levels (1, 4, and 14 g/mL) with five replicates for each concentration were prepared and analyzed. The extraction recovery (R%) was calculated by presenting the ratio of *R*_T_ and *R*_S_ as a percentage, where *R*_T_ referred to the peak area ratio of EGM to IS for QC samples and *R*_S_ was the ratio for the standard solutions. The relative recovery (RR%) of the bioassay method was expressed as the percentage of the measured concentration to the nominal concentration.

Stability studies were performed to assess the EGM stability in rat plasma under various storage and processing conditions, including long‐term storage, short‐term stability, and freeze–thaw cycles. For all stability studies, QC samples were used at three concentrations (1, 4, and 14 g/mL), each with five replicates. Long‐term and short‐term stability were tested by storing plasma samples at −20°C for one month and at room temperature for 24 h, respectively, while freeze–thaw stability was assessed by subjecting the samples to three freeze (−20°C) and thaw (20°C) cycles.

### 2.9. Pharmacokinetic Study of EGM in Rats

The validated SPE‐UPLC analytical method for EGM was employed in the pharmacokinetic study using Wistar rats. Consistent with Section 2.5, all inclusion and exclusion criteria for experimental animals were predefined prior to the commencement of this study. The rats were maintained under controlled conditions (temperature 24 ± 0.5°C, humidity 55 ± 5%, 12 h dark/light cycle) for one week before the experiment to acclimate them to the environment. A total of 40 rats weighing 220 ± 10 g with no overt clinical abnormalities were randomized into 8 groups (*n* = 5 per group) according to blood collection times (0, 0.25, 0.5, 1, 2, 4, 6, and 8 h). On the day of the study, each rat was administered EGM (50 mg/kg) by oral gavage after an overnight fast. The drug was prepared in CMC‐Na (0.5% in water). Blood samples were collected at the specified time points into prelabeled tubes containing Na‐EDTA and were centrifuged at 18,447 × g for 10 min at 4°C. The plasma supernatants were stored at −20°C for further analysis. During the whole experiment, there was no animal death or sample failure. Consequently, all the data of rats were included in the final statistical analysis without any exclusion.

The intragastric dose of EGM was determined based on pharmacokinetic studies of other piperic acid derivatives, including piperine and potassium piperate. Administration of piperine (100 mg/kg) in rats resulted in decreased food intake, reduced body weight, and a pale yellow, swollen stomach. In contrast, rats given 50 mg/kg of potassium piperate remained healthy, and the peak plasma concentration (*C*_max_) was 10 times higher than the LOQ in the UPLC analysis. Therefore, initial dosing of EGM at 10 and 50 mg/kg EGM was conducted to identify the appropriate dose. The chromatogram of plasma samples collected 15 min after intragastric administration of 50 mg/kg EGM exhibited a peak with a higher intensity than the lower limit of detection (LOD). However, the peak intensity of EGM following the 10 mg/kg dose was too low to be easily detected (Figure S6). Therefore, a 50 mg/kg dose was selected for the study. Plasma samples were processed using the SPE method, and EGM concentrations were determined using the previously described UPLC method. The pharmacokinetic data were processed using WINNONLIN software (ver. 5.2).

## 3. Results and Discussion

### 3.1. Structural Characterization of EGM

The crude product was purified by silica gel column chromatography using ethyl acetate: n‐hexane (4:6, v/v) as the MP, obtaining yellow crystals (Figure S1b). The structure of the resulting EGM was verified by NMR, ESI‐MS, and FTIR (Figure S3–S6). ESI‐MS was performed under the following conditions: capillary voltage was +3 kV, source temperature was maintained at 120°C, and desolvation temperature was 450°C. The cone gas and desolvation gas FRs were 100 L/h and 800 L/h, respectively, using nitrogen as both the sheath and auxiliary gas. Positive ionization mode was employed. The HR‐ESI‐MS spectrum exhibited a major (M+H)^+^ peak at m/z 262.01 for EGM (C_14_H_14_O_5_). NMR spectra were acquired on a Bruker Avance NEO 600 NMR spectrometer operating at 600 MHz for ^1^H and 125 MHz for ^13^C. Generally, the exchangeable hydrogen on the hydroxyl group was not observed in the ^1^HNMR. *δ* 2.046 corresponded to the exchangeable hydrogen from water and hydroxyl groups. ^1^HNMR (600 MHz, CDCl_3_) *δ*: 5.94 (*d*, 1H, *J* = 14, CH=CH‐CH=CH), 7.45 (dd, 1H, CH=CH‐CH=CH), 6.74 (dd, 1H, CH‐CH=CH), 6.76 (*d*, 1H, CH=CH‐CH=CH), 6.99 (*s*, 1H, Ar‐7H), 6.93 (*d*, *J* = 7.7, 1H, Ar‐10H), 6.85 (*d*, *J* = 8.2, 1H, Ar‐11H), 5.99 (*s*, 2H, O‐CH_2_‐O), 2.52 (*t*, *J* = 8.82, 2H, CH_2_‐OH), 2.49 (*t*, *J* = 8.76, 2H, O‐CH_2_). ^13^CNMR (125 MHz, CDCl_3_) *δ*: 166.59 (C=O), 148.66 (C_8_), 148.28 (C_9_), 145.7 (C_3_), 140.80 (C_5_), 130.40 (C_6_), 124.29 (C_4_), 123.08 (C_2_), 119.30 (C_11_), 108.52 (C_10_), 105.87 (C_7_), 101.39 (O‐CH_2_‐O), 63.86 (O‐CH_2_), 41.72 (CH_2_‐OH). From the FTIR spectrum (Figure S4), 1701.62 cm^−1^ (C=O absorption band), 1619.48 cm^−1^ (absorption band of conjugated double bonds), 3423.28 cm^−1^ (‐OH absorption band), and 1447.35 cm^−1^ (—CH_2_ absorption band) were observed.

Thus, NMR, MS, and FTIR data analysis confirmed the successful synthesis of EGM, with a 60% yield and 99.2% purity determined by UPLC.

### 3.2. Method Development

In this study, UPLC was employed for the pharmacokinetic analysis of EGM in rat plasma. To optimize the chromatographic conditions, various MPs and FRs were evaluated. As depicted in Figure S7a, using an MP consisting of acetonitrile and acidified water in a 1:1 volumetric ratio resulted in an improved peak shape with minimal tailing and increased peak area. To further optimize the method and mitigate interference in actual plasma samples, the retention time of EGM was adjusted by modifying the FR. (Figure S7b). presents chromatograms for EGM at different FRs of 0.3, 0.25, and 0.2 mL/min. The optimal FR was determined to be 0.2 mL/min, which provided the largest peak area and a retention time of 3.06 min. When the optimized UPLC method was applied to rat plasma samples, an unidentified peak appeared at approximately 2.3 min, which was presumably a relevant metabolite of the investigated compound. However, under the same UPLC conditions, the chromatogram of a pure piperic acid standard (Figure S8) exhibited a distinct retention time of approximately 2.82 min, thereby ruling out the possibility that the unknown peak could be attributed to the hydrolysis product piperic acid. Notably, the unidentified peak at around 2.3 min did not interfere with the determination of EGM. This optimized method was deemed suitable for further pharmacokinetic studies due to its efficiency and selectivity in detecting EGM.

### 3.3. Method Validation

#### 3.3.1. Selectivity

The chromatograms of blank plasma, a quality control (QC) sample of EGM at the lower LOQ mixed with the internal standard (IS, piperine), and a plasma sample taken 15 min postoral administration of EGM are presented in Figure 3. The symmetrical peaks for EGM (retention time 3.05 min) and piperine (retention time 6.26 min) were well‐separated from each other and endogenous plasma components. The accuracy and precision for three parallel analytical samples from five different rat plasma sources were within acceptable limits, with RE 14.6% and RSD 8.6%. This indicates that the detection of both EGM and piperine demonstrated high specificity and selectivity without interference.

**FIGURE 3:**
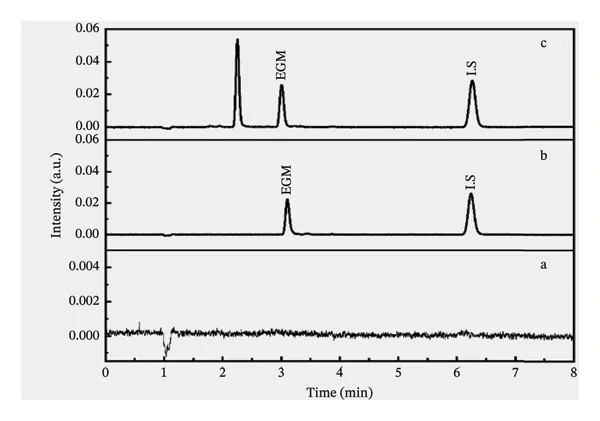
Chromatograms of (a) blank rat plasma; (b) blank rat plasma spiked with EGM and I.S; (c) plasma sample collected at 15 min after an oral administration of 50 mg/kg EGM.

#### 3.3.2. Linearity

The calibration curve (Figure 4) for EGM showed excellent linearity within the 1.0–16.0 g/mL concentration range, with a correlation coefficient (*R* ^2^) of 0.9995. The linear regression equation for EGM was *y* = 0.845 × −0.218 (*R* ^2^ = 0.9995).

**FIGURE 4:**
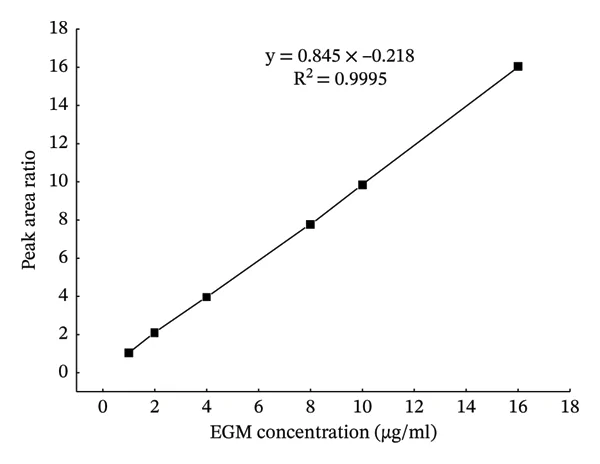
Mean calibration curve of EGM analytical samples (*n* = 6).

#### 3.3.3. Precision and Accuracy

The interday and intraday precision and accuracy were evaluated using QC samples at three concentration levels (1.0, 4.0, and 14.0 g/mL) with five replicates per concentration, as shown in Table 1. Precision was expressed as RSD%, while accuracy was reported as RE%. The summarized results in Table 1 demonstrate that the interday precision ranged between 6.80% and 9.76%, and intraday precision varied between 6.30% and 7.31%, both within the accepted limit of 10% RSD. Similarly, the interday and intraday accuracy ranged from −14.50% to 3.01% and −13.50% to 2.60%, respectively, also meeting the allowed limit of 15% RE.

**TABLE 1:** The validation parameters for the developed method of EGM in rat plasma (*n* = 5).

QC (μg/mL)	Interday (RE%)	Intraday (RE%)	Interday (RSD%)	Intraday (RSD%)	Extraction recovery (Mean ± SD)	Relative recovery (Mean ± SD)	Stability ± RSD%		
							Room temperature (24 h)	Three freeze–thaw cycles	Long term (−20°C, 1 month)
1	2.6	3.01	9.76	6.80	76.56 ± 3.13	102.68 ± 10.02	99.75 ± 2.89	98.99 ± 5.20	99.10 ± 6.32
4	−13.5	−14.5	7.80	7.31	75.32 ± 5.7	86.54 ± 6.84	98.28 ± 5.59	98.12 ± 6.85	99.04 ± 7.18
14	−11.5	−9.36	8.64	6.30	81.93 ± 2.11	88.41 ± 7.62	98.83 ± 7.79	98.69 ± 5.47	99.01 ± 6.89

#### 3.3.4. Recovery

Table 1 presents the relative recovery of the bioanalytical method and the extraction recovery of EGM in rat plasma. The recovery was assessed using QC samples at 1, 4, and 14 g/mL concentrations with five replicates at each level. The mean recovery of both EGM and the IS (piperine) in rat plasma was consistently greater than 75% (RSD < 7.57%), confirming the effectiveness of the extraction process. For the SPE‐UPLC method, the mean recovery was higher than 86% (RSD 9.76%), indicating that the method provided reliable recovery for quantitative analysis.

#### 3.3.5. Stability

The EGM stability in biological matrices was assessed under various conditions, as shown in Table 1. The recovery of EGM QC samples was above 98% with an RSD of less than 10% for both short‐term storage at 25°C for 24 h and long‐term storage at −20°C for one month. These results indicated a high stability of EGM in plasma samples. Furthermore, after three freeze–thaw cycles, the recovery was 98% with an RSD of 6.85%, showing no significant difference from fresh samples. This confirms the stability of EGM under different storage and handling conditions.

### 3.4. Cytotoxicity of EGM in Vitro

Before evaluating the antihyperlipidemic activity of EGM, verifying its biocompatibility is a critical first step. A cell viability test for EGM was conducted on HepG2 cells, as shown in (Figure 5), where cell viability exceeded 90% at concentrations up to 150 μg/mL and remained above 70% up to 300 μg/mL, indicating low cytotoxicity of EGM at physiologically relevant concentrations. At higher concentrations, a gradual, concentration‐dependent decrease in cell viability was observed, with viability reduced to approximately 55% at 500 μg/mL. These results confirm the favorable safety profile of EGM in hepatic cells.

**FIGURE 5:**
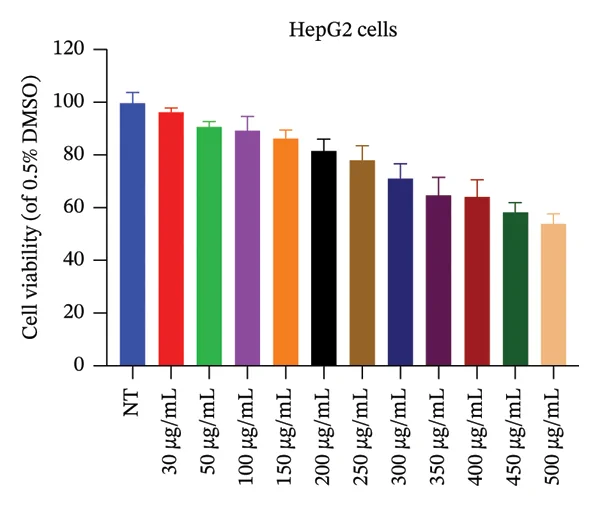
Viabilities of HepG2 cells incubated with EGM at different concentrations for 24 h.

### 3.5. Antihyperlipidemic Activity

Following 15 days on a high‐lipid diet, the HFD group showed significantly higher plasma levels of TC, TG, LDL‐C, and GLU, along with a lower level of HDL‐C compared to the control group (Figure 6 and Table 2), establishing a hyperlipidemic rat model. The hyperlipidemic rats were treated with EGM and Simvastatin. As shown in Figure 6 and Table 2, EGM significantly decreased TC (Figure 6a), TG (Figure 6b), LDL‐C (Figure 6c), and GLU (Figure 6d) levels while slightly increasing HDL‐C (Figure 6e) levels. These results demonstrate that EGM has the potential to effectively reduce blood lipid levels, at least in animal models. Moreover, both EGM and Simvastatin had significant lipid‐lowering activities, with EGM showing better results in reducing TC, GLU, and LDL‐C and increasing HDL‐C compared to Simvastatin.

**FIGURE 6:**
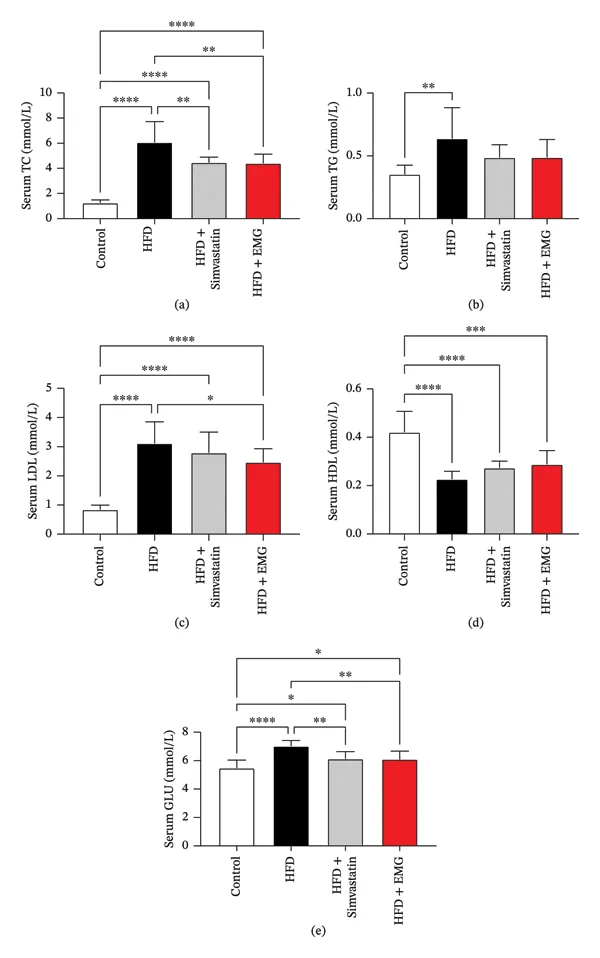
Effect of EGM and Simvastatin on lipid parameters of rats in vivo (*n* = 10). (a) TC; (b) TG; (c) LDL; (d) HDL; (e) GLU.

**TABLE 2:** The TC, TG, HDL, LDL, GLU content in rat serum (unit: mmol/L).

Group	Dose (mg/kg)	TC (mmol/L)	TG (mmol/L)	LDL‐C (mmol/L)	HDL‐C (mmol/L)	GLU (mmol/L)
Control	—	1.25 ± 0.24^∗∗∗∗^	0.35 ± 0.07^∗∗^	0.85 ± 0.15^∗∗∗∗^	0.42 ± 0.08^∗∗∗∗^	5.46 ± 0.57^∗∗∗∗^
HFD	—	6.06 ± 1.05	0.64 ± 0.25	3.11 ± 0.70	0.23 ± 0.05	7.00 ± 0.541
HFD + Simvastatin	5.6	4.47 ± 0.42^∗∗^	0.48 ± 0.10	2.79 ± 0.68	0.27 ± 0.03	6.11 ± 0.53^∗∗^
HFD + EGM	5.6	4.40 ± 0.73^∗∗^	0.49 ± 0.11	2.47 ± 0.44^∗^	0.29 ± 0.05	6.08 ± 0.59^∗∗^

*Note:* Values are expressed as mean ± standard deviation (SD). All statistical comparisons were performed relative to the HFD group.

^∗^ *p* < 0.05.

^∗∗^ *p* < 0.01.

^∗∗∗∗^ *p* < 0.0001 vs. HFD group.

The antihyperlipidemic activity of EGM is closely related to its structural characteristics, which retain the 1,2‐methylenedioxyphenyl group and conjugated double bond fragments originally present in piperine. These structural fragments serve as essential pharmacophores responsible for lipid regulatory activity (https://doi.org/10.13822/j.cnki.hxsj.2022.0260). Mechanistically, EGM exerts lipid‐lowering effects through multiple synergistic pathways: it may activate PPARα/*γ* to accelerate fatty acid oxidation (https://doi.org/10.1016/j.bbrc.2025.151895), inhibit HMG‐CoA reductase to reduce cholesterol synthesis (https://doi.org/10.1080/03639045.2019.1569034), regulate ABCA1/G1 to promote reverse cholesterol transport (https://doi.org/10.13484/j.nmgdxxbzk.20160107), and relieve oxidative stress (https://doi.org/10.1016/j.carbpol.2007.06.014).

### 3.6. Pharmacokinetic Study

The pharmacokinetic profile of EGM in male Wistar rats was successfully determined using the above‐mentioned SPE‐UPLC method. The concentrations of EGM in plasma were analyzed, and the pharmacokinetic analysis was performed using WINNONLIN software (ver. 5.2) [20]. The mean concentration‐time curve of EGM (Figure 7) following a single oral dose (50 mg/kg) exhibited a bimodal absorption phase (likely due to enterohepatic circulation or gastric emptying delay) and a monoexponential elimination phase, with no distinct biphasic decline typical of multicompartment models. Given the lack of biphasic decline in the elimination phase and the principle of parsimony, the one‐compartment model was selected for PK fitting, which provided stable parameters and good goodness‐of‐fit. The pharmacokinetic parameters are given in Table 3. The data demonstrated that EGM was absorbed rapidly after oral administration and showed a slow clearance from the bloodstream in Wistar rats, indicating prolonged systemic exposure.

**FIGURE 7:**
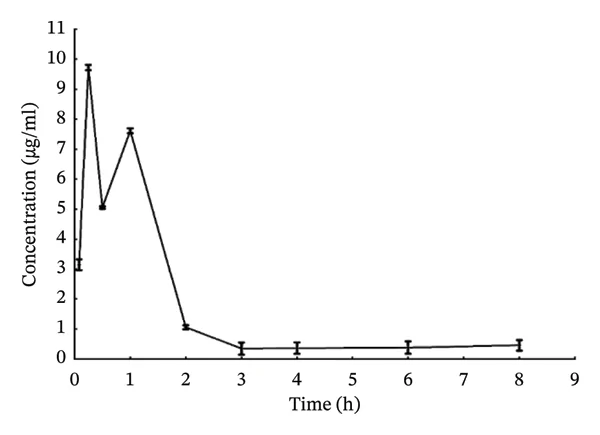
Mean plasma concentration–time curves of EGM after oral single dose of 50 mg/kg in male Wistar rats. (The data points are means, and positive error bars are SD of six animals).

**TABLE 3:** The pharmacokinetic parameters of EGM after oral single dose of 50 mg/kg to male Wistar rats (*n* = 5, mean ± SD).

PK parameters	Unit	Values (mean ± SD)
*t*_1/2_	h	1.46 ± 0.67
*T*_max_	h	0.25
*C*_max_	ng/mL	9780 ± 42.87
AUC_0–∞_	ng·h/mL	715,530 ± 522.4
MRT	h	1.98 ± 0.29
CL_F	mL/h	4192.8 ± 458.1
Vd/F	L/kg	40.18

### 3.7. Limitations and Future Perspectives

This study reports the design and synthesis of EGM compounds, along with the evaluation of their antihyperlipidemic activity and a pharmacokinetic study. To enhance this activity, a novel EGM analog was designed and synthesized. However, a limitation of this work is that microfluidic technology and nanotechnology have not yet been fully integrated into drug delivery systems [22, 23], a limited sample cohort, a lack of chronic toxicity evaluations, and no elucidation of the underlying molecular mechanisms [24, 25]. To address these shortcomings, future research will expand animal sample sizes across multiple strains, conduct 12‐week chronic toxicity studies to assess hepatic function, hematological parameters, and histopathology, perform in‐depth mechanistic validation via RT‐qPCR and surface plasmon resonance (SPR) assays, and leverage microfluidic technology to precisely control the fluid behavior of EGM at the micro and nanoscale.

## 4. Conclusions

This study demonstrated that EGM, which contains 1,2‐methylenedioxybenzene and hydroxyl groups, is significantly effective in reducing blood lipid levels in Wistar rats. In antihyperlipidemic studies, EGM demonstrated promising performance in reducing TC, GLU, and LDL‐C, as well as elevating HDL‐C in this preclinical model. A sensitive, rapid, and accurate UPLC method was established and validated to determine EGM in rat plasma after an oral single dose (50 mg/kg). The pharmacokinetic study indicated that EGM is rapidly absorbed and slowly cleared, leading to prolonged systemic exposure. These findings suggest that piperinic acid derivatives, including EGM, have the potential to be further investigated as novel active pharmaceutical ingredients (APIs) with antihyperlipidemic properties [26].

**Nomenclature**
EGM: Ethylene glycol monopiperate
SPE: Solid‐phase extraction
UPLC: Ultra performance liquid chromatography
NMR: Nuclear magnetic resonance spectroscopy
IR: Infrared radiation
MS: Mass spectrometry
IS: Internal standard
HFD: High‐fat diet
TC: Total cholesterol
LDL‐C: Low‐density lipoprotein cholesterol
HDL‐C: High‐density lipoprotein cholesterol
TG: Triglycerides
GLU: Blood glucose
LOQ: Limit of quantification
LOD: Limit of detection
QC: Quality control
RE: Relative error
RSD: Relative standard deviation
CMC‐Na: Sodium carboxymethyl cellulose
SPE: Solid‐phase extraction

## Funding

This study was supported by the Natural Science Foundation of Inner Mongolia Autonomous Region, Grant No. 2025FX032 HM‐TD‐202516, YAU202507712.

## Ethics Statement

The animal study protocol was approved by the Ethics Committee of Hohhot Minzu College (protocol code HMC 20250309 and March 09, 2025).

## Conflicts of Interest

The authors declare no conflicts of interest.

## Supporting Information

Additional supporting information can be found online in the Supporting Information section.

## Supporting information


**Supporting Information** The following supporting information can be downloaded at https://www.mdpi.com/article/doi/s1. Figure S1: The optical microscope images of (a) piperic acid; (b) EGM; S2: The chromatogram of EGM in plasma after being treated with LLE and SPE. Figure S3: ^1^HNMR of the synthesized EGM in CDCl_3_ (600 MHz, CDCl_3_); Figure S4: ^13^CNMR of the synthesized EGM in CDCl_3_ (125 MHz, CDCl_3_); Figure S5: ESI‐MS spectrum of the synthesized EGM; Figure S6: FTIR spectrum of the synthesized EGM; Figure S7: The chromatograms of EGM (a) using mobile phases with different proportions; (b) using different flow rates; Figure S8: The chromatogram of piperic acid (black line) and plasma sample collected at 15 min after an oral administration of 50 mg/kg EGM (red line); Figure S9: The chromatogram of the rat plasma sample collected at 15 min after an oral administration of different doses.

## Data Availability

The data that support the findings of this study are available from the corresponding author upon reasonable request.
