# Supplementary material for: Design and Evaluation of Ethylene Glycol Monopiperate as a Novel Antihyperlipidemic Prodrug: Synthesis, Antihyperlipidemic Activity, and Pharmacokinetics
Source: J Anal Methods Chem. 2026 Oct 27;2026:3258325. doi: 10.1155/jamc/3258325 (PMC13711490; doi:10.1155/jamc/3258325)
Supplement: Supplementary file 1 [file JAMC-2026-3258325-s001.docx]

Supplemental

|  |  |
| --- | --- |
| (**a**) | (**b**) |

**Figure S1.** The optical microscope images of (a) Piperic acid; (b) EGM

**Figure S2.** The chromatograms of EGM in plasma after being dealt with LLE and SP

**Figure S3.** ^1^H NMR of the synthesized EGM in CDCl_3_ (600MHz, CDCl_3_).

**Figure S4.** ^13^C NMR of the synthesized EGM in CDCl_3_ (125MHz, CDCl_3_).

**Figure S5.** ESI-MS spectrum of the synthesized EGM.

**Figure S6.** FTIR spectrum of the synthesized EGM.

|  |  |
| --- | --- |
| (**a**) | (**b**) |

**Figure S7.** The chromatogram of EGM (a) using mobile phases with different proportions; (b) using different flow rates.

**Figure S8** The chromatogram of piperic acid (black line) and plasma sample collected at 15 min after an oral administration of 50 mg/kg EGM (red line).

**Figure S9.** The chromatogram of the rat plasma sample collected at 15 min after oral administration of different doses.
